# Antibody Responses to the Conserved *Plasmodium falciparum* Vacuolar Sorting Protein 29 in the Brazilian Amazon

**DOI:** 10.3390/pathogens15070691

**Published:** 2026-06-30

**Authors:** Juliana Aline Souza Lemos, Barbara de Oliveira Baptista, Carolina de Souza Faria Pereira, Hugo Amorim dos Santos de Souza, Jenifer Peixoto de Barros, Rodrigo Medeiros Martorano, Rodrigo Nunes Rodrigues-da-Silva, Evelyn Kety Pratt Riccio, Dave Richard, Paulo Renato Rivas Totino, Josué da Costa Lima-Junior, Cláudio Tadeu Daniel-Ribeiro, Lilian Rose Pratt-Riccio

**Affiliations:** 1 Laboratório de Pesquisa em Malária, Instituto Oswaldo Cruz (IOC), Fundação Oswaldo Cruz (Fiocruz), Rio de Janeiro 21040-900, RJ, Brazil; julianalemos@aluno.fiocruz.br (J.A.S.L.); barbara.baptista@ioc.fiocruz.br (B.d.O.B.); carolinasouzafaria@gmail.com (C.d.S.F.P.); hugosouza@aluno.fiocruz.br (H.A.d.S.d.S.); jeniferbarros@aluno.fiocruz.br (J.P.d.B.); ericciovazoler@hotmail.com (E.K.P.R.); prtotino@ioc.fiocruz.br (P.R.R.T.); malaria@fiocruz.br (C.T.D.-R.); 2 Centro de Pesquisa, Diagnóstico e Treinamento em Malária (CPD-Mal), Fiocruz e Secretaria de Vigilância em Saúde e Ambiente, Ministério da Saúde, Rio de Janeiro 21040-900, RJ, Brazil; 3 Laboratório de Doenças Infecciosas na Amazônia Ocidental, Universidade Federal do Acre—Campus Floresta (UFAC), Cruzeiro do Sul 69895-000, AC, Brazil; rodrigo.martorano@ufac.br; 4 Laboratório de Hantaviroses e Rickettsioses, Instituto Oswaldo Cruz (IOC), Fundação Oswaldo Cruz (Fiocruz), Rio de Janeiro 21040-900, RJ, Brazil; nunes@ioc.fiocruz.br; 5 Department of Microbiology-Infectious Diseases and Immunology, Faculty of Medicine, Université Laval, Quebec City, QC G1V 4G2, Canada; dave.richard@crchudequebec.ulaval.ca; 6 CHU de Québec-Université Laval Research Centre, Université Laval, 2705 Boul. Laurier, Quebec City, QC G1V 4G2, Canada; 7 Centre de Recherche en Infectiologie, Université Laval, 2705 Boul. Laurier, Quebec City, QC G1V 4G2, Canada; 8 Laboratório de Imunoparasitologia, Instituto Oswaldo Cruz (IOC), Fundação Oswaldo Cruz (Fiocruz), Rio de Janeiro 21040-900, RJ, Brazil; josue@ioc.fiocruz.br

**Keywords:** *P. falciparum*, PfVPS29, immune response, malaria, retromer complex

## Abstract

Vacuolar Protein Sorting 29 (VPS29) is a highly conserved subunit of the retromer complex, which mediates retrograde transport from endosomes to the Golgi apparatus and plays a critical role in membrane trafficking, protein recycling, and organelle biogenesis. In *Plasmodium falciparum*, the retromer has been implicated in the formation of apical organelles essential for parasite invasion and replication. In this study, we investigated naturally acquired antibody responses to *P. falciparum* VPS29 (PfVPS29) and the genetic diversity of the *vps29* gene in isolates from three malaria-endemic areas of the Brazilian Amazon. Naturally acquired responses to PfVPS29 were evaluated by ELISA in 533 individuals, and genetic diversity was assessed in 62 *P. falciparum* isolates. Only 17% of participants displayed IgG reactivity, whereas 73.5% showed IgM responses, indicating limited IgG acquisition but a predominant IgM profile associated with recent or ongoing exposure. IgG subclass analysis revealed a predominance of cytophilic IgG1 and IgG3 among responders. IgM responses were significantly boosted during *P. falciparum* infection. Sequence analysis revealed no polymorphisms among Brazilian isolates, and comparison with global datasets confirmed the high conservation of the PfVPS29 coding sequence. Together, these findings show that PfVPS29 is a highly conserved intracellular protein that elicits an atypical humoral response dominated by IgM, with limited class switching to IgG, like other conserved or repetitive malaria antigens. These results highlight PfVPS29 as an example of a conserved intracellular antigen that induces non-classical humoral responses in naturally exposed populations.

## 1. Introduction

Malaria, caused by *Plasmodium* parasites and transmitted by *Anopheles* mosquitoes, remains one of the most devastating infectious diseases worldwide, with the highest burden occurring in sub-Saharan Africa. Among the five *Plasmodium* species that infect humans, *Plasmodium falciparum* is the most virulent and accounts for the majority of malaria-related morbidity and mortality. In 2023, the World Health Organization (WHO) reported 263 million cases and approximately 597,000 deaths [1]. Artemisinin-based combination therapies (ACTs) are the current first-line treatment for *P. falciparum* malaria [2]. However, ACT-resistant *P. falciparum* isolates have been detected in several regions. First reported in Western Cambodia nearly two decades ago [3], resistance has since spread across the Greater Mekong Subregion, India, and Papua New Guinea [4,5,6,7,8]. In addition, artemisinin partial resistance has independently emerged in several African countries, including Rwanda, Uganda, Tanzania, and Eritrea [9,10]. Given these challenges, developing effective vaccines is essential to malaria control efforts, as vaccination has the potential to reduce morbidity and mortality beyond what can be achieved through chemotherapeutic interventions alone. Although two vaccines, RTS,S/AS01 and R21/Matrix-M, have now been licensed for use, their modest and relatively short-lived efficacy underscores the need to identify additional antigens. Moreover, current vaccination recommendations are limited to young children, and guidelines for older children, adolescents, and adults have not yet been established, further highlighting the need for broader and more durable vaccine solutions [11].

Within this context, the retromer complex has gained increasing attention for its critical role in endosomal protein sorting and trafficking. This highly conserved multiprotein assembly is essential for recycling transmembrane receptors and transporting cargo from endosomes to the trans-Golgi network or plasma membrane. In most eukaryotic cells, the core retromer complex consists of the VPS26, VPS29, and VPS35 (Vacuolar Protein Sorting) subunits, which form a stable trimer responsible for cargo recognition and retrieval, often in association with sorting nexins (SNXs) that mediate membrane curvature and tubule formation. Among these components, VPS29 is a particularly conserved core subunit. Immunolocalization studies have shown that PfVPS29 is expressed throughout the asexual stages of *P. falciparum*, with the highest abundance in trophozoites [12]. This 21.7 kDa monomeric protein shares structural similarities with metallophosphoesterases, although it lacks catalytic activity and functions mainly as a scaffold, binding the C-terminal domain of VPS35 to stabilize retromer interactions with accessory proteins [12,13,14]. While the precise function of VPS29 remains uncharacterized in *Plasmodium* spp., studies in *Toxoplasma gondii* show that retromer disruption via TgVps35 knockdown impairs the biogenesis of secretory organelles and parasite morphology [15], indicating that this trafficking pathway is essential in Apicomplexans. The extensive conservation of VPS29 across species [14,16] is consistent with strong functional constraints and suggests limited tolerance for mutations, supporting its relevance for studies of parasite biology and host immune recognition.

Although PfVPS29 is recognized as an essential component of the retromer complex, its antigenic properties and genetic variability in *P. falciparum* remain poorly characterized. Determining whether PfVPS29 elicits specific immune responses and whether genetic polymorphisms affect its antigenicity or population distribution may provide valuable insights into parasite adaptation and immune evasion. Therefore, this study aimed to characterize naturally acquired humoral responses to PfVPS29 in individuals living in malaria-endemic regions of Brazil and to assess the genetic polymorphism of the vsp29 gene in *P. falciparum* field isolates. Together, these analyses contribute to a broader characterization of PfVPS29 and help clarify its potential relevance in parasite biology and immune recognition.

## 2. Materials and Methods

### 2.1. Study Area and Sample Collection

The cross-sectional cohort study was conducted between June and August of 2016, 2018, and 2023 in three malaria-endemic regions of the Brazilian Amazon: Cruzeiro do Sul (07°37′50″ S/72°40′13″ W) and Mâncio Lima (07°36′49″ S/72°53′47″ W), both high-risk areas located in the Juruá Valley (Acre State), the main hotspot for *P. falciparum* malaria in Brazil, and Guajará (07°32′45″ S/72°35′02″ W), a medium-risk area in Amazonas State. Together, these municipalities account for approximately 52.6% of *P. falciparum* malaria cases in Brazil and exhibit some of the highest Annual Parasite Incidence (API) rates, defined as the number of positive blood slides per 1000 inhabitants. Due to the increase in *P. falciparum* cases in other municipalities, this number decreased to 24.1% in 2018. According to the Brazilian Ministry of Health, areas with an API ≥ 50 are considered high risk. In 2016, the reported API values for *P. falciparum* were 85.3 in Cruzeiro do Sul, 67.3 in Mâncio Lima, and 43.3 in Guajará.

A total of 533 malaria-exposed individuals were enrolled in this study, including 257 from Cruzeiro do Sul (CZS), 151 from Mâncio Lima (ML), and 125 from Guajará (GJ). Written informed consent was obtained from all adult participants or from the parents or guardians of child participants. All individuals who consented also completed an epidemiological survey. To assess the degree of malaria exposure, participants provided personal and clinical information, including age, time of residence in the endemic area, number of previous malaria episodes, time since the last infection, use of malaria prophylaxis, and presence of symptoms.

For immunological analyses, peripheral blood samples were collected in heparin tubes. After centrifugation (350× *g*, 10 min), plasma was separated and stored at −20 °C. For molecular analyses, blood samples were collected in EDTA tubes, centrifuged under the same conditions, and the cell pellet was preserved at −20 °C in glycerolyte solution (1:2). Thin and thick blood smears were examined under oil immersion at 1000× magnification, with 200 fields screened for parasitological evaluation. Thin blood smears from positive samples were further inspected for species identification. Parasitemia was quantified in thick blood smears as the number of parasites per microliter (µL) of blood. Parasites were counted against 500 white blood cells (WBCs), and parasite density was calculated assuming a standard leukocyte count of 8000 WBCs/µL as previously described [17]. To increase diagnostic sensitivity, molecular analyses were performed on all samples. DNA was extracted using the QIAamp DNA blood mini kit (Qiagen, Germantown, MD, USA) according to the manufacturer’s instructions. Polymerase Chain Reaction (PCR) targeting *Plasmodium* genus-specific and species-specific (*P. falciparum* and *P. vivax*) primers was carried out as previously described [18]. Participants who tested positive for *P. vivax* and/or *P. falciparum* at the time of blood collection received treatment according to the chemotherapeutic guidelines of the Brazilian Ministry of Health [19].

Serum samples from individuals with no history of malaria were included as area controls (CO group, n = 54). All participants in this group tested negative for malaria by thick blood smear examination and PCR. Additionally, non-endemic control samples were obtained from 40 laboratory staff members in Rio de Janeiro, Brazil, who had no history of malaria and had never visited malaria-transmission areas (Rio de Janeiro Controls).

### 2.2. Recombinant Proteins and Antibody Assays

The recombinant PfVPS29 protein, derived from the *P. falciparum* 3D7 reference strain (UniProt ID: Q8IM27), was commercially produced in Escherichia coli (BL21(DE3) strain) by GenOne Biotechnologies (Rio de Janeiro, Brazil). The coding sequence was cloned into an expression vector containing a C-terminal His-tag to enable affinity purification. Protein expression was induced with 0.5 mM isopropyl β-D-1-thiogalactopyranoside (IPTG) at 37 °C for 4 h. Following induction, bacterial cells were harvested and lysed by ultrasonication (3 s pulses with 6 s intervals, for a total of 5 min) in lysis buffer containing 50 mM Tris-HCl and 300 mM NaCl (pH 8.0). Solubility analysis indicated that the recombinant protein was present in the soluble fraction. The clarified lysate was purified by immobilized metal affinity chromatography (IMAC) using Ni-NTA 6FF resin, consistent with the presence of the His-tag. The column was equilibrated with PBS (pH 7.4), and non-specifically bound proteins were removed by washing with PBS containing 50 mM imidazole. The recombinant protein was subsequently eluted with PBS containing 500 mM imidazole. Eluted fractions containing PfVPS29 were pooled and dialyzed against PBS (pH 7.4) to remove excess imidazole and ensure buffer exchange. The purified recombinant protein was supplied lyophilized in phosphate-buffered saline (PBS; pH 7.4). Protein purity (>90%) was assessed by sodium dodecyl sulfate–polyacrylamide gel electrophoresis (SDS–PAGE), followed by Coomassie blue staining, according to the manufacturer’s quality control report (Supplementary Figure S1). Protein concentration was determined using the Bradford assay. Given the use of a bacterial expression system, potential contamination with lipopolysaccharide (LPS) cannot be excluded. Endotoxin levels were not reported in the manufacturer’s documentation. However, all immunoassays were performed under identical experimental conditions, minimizing potential bias in comparative analyses.

Microtiter 96-well plates (Maxisorp, NUNC, Roskilde, Denmark) were coated with 0.5 µg/mL of recombinant protein in carbonate-bicarbonate buffer, pH 9.6, 100 µL/well, and incubated overnight at 4 °C. Plates were washed with phosphate-buffered saline containing 0.05% Tween 20 (PBST), and blocked with 5% powdered milk in PBST for 1 h at 37 °C. Serum samples diluted 1:50 in dilution buffer (1% powdered milk in PBST) were added in duplicate, and the plates were incubated for 2 h at 37 °C. After washing, 100 µL of peroxidase-conjugated mouse anti-human IgG or IgM (Sigma, St. Louis, MO, USA), diluted to 1:2000 in dilution buffer, was added, followed by incubation for 1 h at 37 °C. For IgG subclass detection, plates were incubated with peroxidase-conjugated mouse anti-human IgG1, IgG2, IgG3, or IgG4 (Clones: 4E3, 31-7-4, HP6050, and HP6025 for IgG1, IgG2, IgG3, and IgG4, respectively; SouthernBiotech, Birmingham, AL, USA), diluted 1:1000 in dilution buffer, for 1 h at 37 °C. After a final wash, 100 µL of substrate solution containing 0.4 mg/mL orthophenylenediamine (OPD, Sigma, St. Louis, MO, USA) and 30%H2O2 (Sigma, St. Louis, MO, USA) in citrate-phosphate buffer (24 mM citric acid, Sigma, St. Louis, MO, USA, and 51 mM dibasic sodium phosphate, Sigma, St. Louis, MO, USA), pH 5.0, was added. The plates were incubated for 5 min at room temperature in the dark, and the reaction was stopped with 50 µL/well of 2N H2SO4 (Sigma, St. Louis, MO, USA). The optical density (OD) was measured at 492 nm using a SpectraMax 250 ELISA reader (Molecular Devices, Sunnyvale, CA, USA). Serum from five individuals living in non-endemic areas (Rio de Janeiro) were used to establish the normal range for each assay. The cut-off values were determined as the mean OD of the non-endemic control samples plus three standard deviations (SD). The results were expressed as semi-quantitative Reactivity Indices (RIs), calculated by dividing each sample’s OD mean by the respective cut-off value. Individuals with RI > 1.1 were classified as responders.

### 2.3. DNA Extraction and PCR Amplification

Genomic DNA was extracted from cryopreserved blood samples using the QIAamp DNA blood mini kit (Qiagen, Germantown, MD, USA), according to the manufacturer’s instructions, and stored at −20 °C until use. Specific primers for the *vps29* gene were designed based on the *P. falciparum* 3D7 reference sequence (accession number PF3D7_1406700) available in the NCBI database and chemically synthesized for PCRs and DNA sequencing by GenOne Biotechnologies (Rio de Janeiro, Brazil). Amplification of the pfvps29 gene fragment was performed by conventional PCR using the designed primers: forward 5′-AGATGAGTGGGAAATTGGAAG′ and reverse 5′-GACGTGTAAAAGAGATGTTGGA-3. Reactions were carried out in a ProFlex PCR system (Applied Biosystems, Foster City, CA, USA) in a total volume of 50 μL, containing 4 μL of genomic DNA, 1.5 μL of each primer at 10 pmol/μL concentration, 12 μL of HOT FIREPol^®^ Blend Master Mix (Solis BioDyne, Tartu, Estonia), and 31 μL of Nuclease-free water. The cycling conditions were: initial denaturation at 95°C for 12 min; 35 cycles of 95 °C for 40 s, 58 °C for 40 s, and 72 °C for 1 min and 30 s; followed by a final extension at 72 °C for 7 min. Each reaction included two negative controls (a no-template control and a sample containing DNA from a clinical *P. vivax* isolate) and one positive control consisting of DNA extracted from an in vitro culture of the *P. falciparum* W2 strain. PCR products were analyzed by electrophoresis on a 1.5% agarose gel prepared in 1xTAE buffer (0.04 M TRIS-acetate, 1 mM EDTA) containing 0.5 μg/mL ethidium bromide (Sigma, St. Louis, MO, USA). Bands were visualized under ultraviolet (UV) illumination, and fragment sizes were estimated using the GeneRuler 1000 bp Plus DNA Ladder (Thermofisher Scientific, Waltham, MA, USA).

### 2.4. Sequencing and Polymorphism Analysis

PCR products were purified using the Wizard SV Gel and PCR Clean-up System kit (Promega, Madison, WI, USA) according to the manufacturer’s instructions. Sequencing reactions were performed for each primer using the BigDye™ Terminator v3.1 Cycle Sequencing Kit (Applied Biosystems™) with 5–50 ng of purified PCR products. The resulting fragments were analyzed on a 3730 xl DNA Analyzer (Applied Biosystems). Forward and reverse reads were assembled and quality-checked in SeqMan v.7.0.0 (DNASTAR, v10.0 software package, Lasergene, Madison, WI, USA) using default parameters, followed by manual inspection of chromatograms. A minimum Phred quality score of 20 (base-call accuracy ≥ 99%) was required for all positions. Edited sequences were aligned in MEGA version 7.0 using the Clustal X2 version 2.1 algorithm to identify polymorphisms relative to the *pfvps29* sequence from the 3D7 strain (PlasmoDB: PF3D7_1406700). For a global comparative analysis, full-length pfvps29 nucleotide sequences were retrieved from GenBank through BLAST (NCBI BLAST, version 2.13.0+, NCBI, Bethesda, MD, USA) searches and from PlasmoDB and subsequently aligned to assess genetic variability.

### 2.5. Statistical Analysis

All statistical analyses were performed using Prism 10.0 for Mac (GraphPad Software, Inc., San Diego, CA, USA). The distribution of variables was assessed using the one-sample Kolmogorov–Smirnov test. Fisher’s exact test was used to compare the frequency of responders between groups. Differences in continuous variables were analyzed using the Kruskal–Wallis test followed by Dunn’s multiple-comparison test with adjusted *p*-values, or the Mann–Whitney U test for pairwise comparisons, as indicated in the figure legends. Correlations between variables were evaluated using Spearman’s rank correlation coefficient. Correlation analyses were considered exploratory, and no correction for multiple comparisons was applied. All statistical tests were two-sided, and a *p*-value ≤ 0.05 was considered statistically significant.

## 3. Results

### 3.1. Population Characteristics

The studied population comprised 533 individuals living in three malaria-endemic areas of the Brazilian Amazon, with ages ranging from 12 to 92 years (median: 31; IQR: 21–45) and a balanced sex distribution (47.6% female, 52.4% male) (Table 1). Nearly all participants (99%) reported lifelong exposure to malaria (median: 29 years; IQR: 20–44), and 90.2% had experienced at least one malaria episode. Among individuals who could recall the infecting parasite, most reported previous infections exclusively by *P. vivax* or both *P. vivax* and *P. falciparum*, with *P. vivax* being the most common. The number of past malaria episodes varied widely (0–264; median: 10; IQR: 3–20), as did the time since the last infection (0–864 months; median: 12; IQR: 2–60). At the time of blood collection, 90 (16.9%) individuals were infected with *P. falciparum*, 173 (32.5%) were infected with *P. vivax*, and only one individual (0.19%) presented a simultaneous *P. falciparum* and *P. vivax* infection, as determined by microscopic examination and subsequently confirmed by PCR.

For comparison, a control (CO) group included residents from Cruzeiro do Sul (CZS), Mâncio Lima (ML), and Guajará (GJ) who reported no previous malaria episodes and had neither symptoms nor detectable parasitemia at the time of sampling. Compared with malaria-exposed participants, individuals in the CO group were younger (median: 21 years; IQR: 20–24), had shorter time of residence in endemic areas (median: 21 years; IQR: 19–23), and were predominantly females (59%).

### 3.2. Antibody Response Against PfVPS29

**Figure 1:**
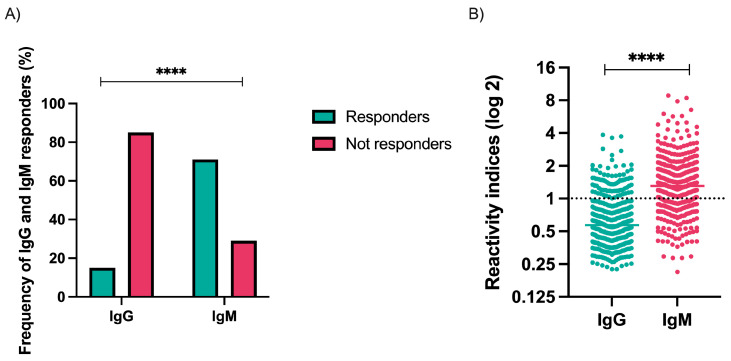
Humoral immune responses to recombinant PfVPS29. (**A**,**B**) Frequency (**A**) and reactivity indices (**B**) of IgG and IgM antibodies recognizing recombinant PfVPS29. Each point represents an individual value (n = 533). Dashed lines indicate the cutoff for positivity (reactivity index > 1). Lines represent medians. Frequencies of responders were compared using Fisher’s exact test, and reactivity indices were compared using the Mann–Whitney test. **** *p* < 0.0001.

The naturally acquired immune response to PfVPS29 remains experimentally unexplored. To address whether PfVPS29 is naturally targeted by humoral responses in malaria-exposed individuals, IgG and IgM reactivity against the recombinant protein was measured in plasma samples from individuals living in the Brazilian Amazon. Only 15% of participants displayed detectable IgG antibodies, whereas a significant proportion of them (71%) showed IgM reactivity (*p* ≤ 0.0001) (Figure 1A). Among IgG responders, reactivity indices ranged from 1.01 to 3.85 (mean: 1.55 ± 0.56) (*p* ≤ 0.0001), while IgM responders displayed a broader range of reactivity (1.1–8.86; mean: 1.88 ± 1.0) (*p* ≤ 0.0001) (Figure 1B).

To investigate whether antibody responses were associated with host-related factors, correlations with epidemiological variables, including age, sex, time of residence in endemic areas, number of reported previous malaria episodes, time elapsed since the last malaria episode, and infection history, were analyzed. No associations were observed between the frequency of IgG responders and any of these variables. Spearman’s rank correlation analysis revealed statistically significant positive correlations between IgG antibody levels and age (*p* = 0.006, r = 0.119), time of residence in endemic areas (*p* = 0.014, r = 0.108), and number of previous malaria episodes (*p* = 0.033, r = 0.096), whereas no significant correlations were detected with the remaining variables. In contrast, IgM antibody levels were negatively correlated with age (*p* < 0.0001, r = −0.202), time of residence in endemic areas (*p* = 0.0002, r = −0.163), and time elapsed since the last malaria infection (*p* = 0.0003, r = −0.165). All statistically significant correlations were characterized by low Spearman correlation coefficients (r ≤ 0.202). A weak but statistically significant positive correlation was also observed between IgG and IgM antibody levels (*p* = 0.0019, r = 0.13), indicating only a minimal association between the two antibody classes.

**Figure 2:**
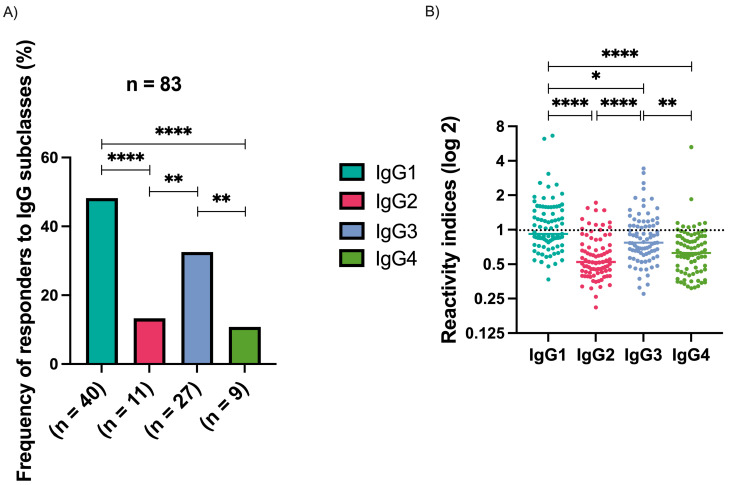
Humoral immune responses to recombinant PfVPS29. (**A**,**B**) Frequency (**A**) and reactivity indices (**B**) of IgG subclasses against PfVPS29 (n = 83). Frequencies were compared using pairwise Fisher’s exact tests. IgG2 vs. IgG3 ** *p* < 0.005, IgG3 vs. IgG4 ** *p* < 0.001, **** *p* < 0.0001. Reactivity indices were compared using the Kruskal–Wallis test followed by Dunn’s multiple-comparison test with adjusted *p*-values. * *p* = 0.037, ** *p* = 0.003, **** *p* < 0.0001.

To characterize the balance between cytophilic and non-cytophilic antibody responses to PfVPS29, we analyzed the distribution and magnitude of IgG subclasses among IgG-positive individuals. PfVPS29 was predominantly recognized by the cytophilic subclasses IgG1 and IgG3. IgG1 was the most prevalent subclass, detected in 48.1% of responders, followed by IgG3 at 32.5%, IgG2 at 13.2%, and IgG4 at the lowest frequency at 10.8%. The frequency of IgG1 responders was significantly higher compared to that of IgG2 (*p* < 0.0001) and IgG4 (*p* < 0.0001). Likewise, the frequency of IgG3 was significantly higher compared to that of IgG2 (*p* = 0.0052) and IgG4 (*p* = 0.012) (Figure 2A). Regarding the magnitude of antigen-specific IgG subclass responses, IgG1 reactivity indices (RIs) were significantly higher than IgG2 (*p* ≤ 0.0001), IgG3 (*p* = 0.0372), and IgG4 (*p* ≤ 0.0001) RIs. Additionally, IgG3 RIs were significantly higher than IgG2 (*p* ≤ 0.0001) and IgG4 RIs (*p* = 0.0035) (Figure 2B). Moreover, IgG2 antibody levels were positively correlated with the time of residence in endemic areas (*p* = 0.034, r = 0.227).

**Figure 3:**
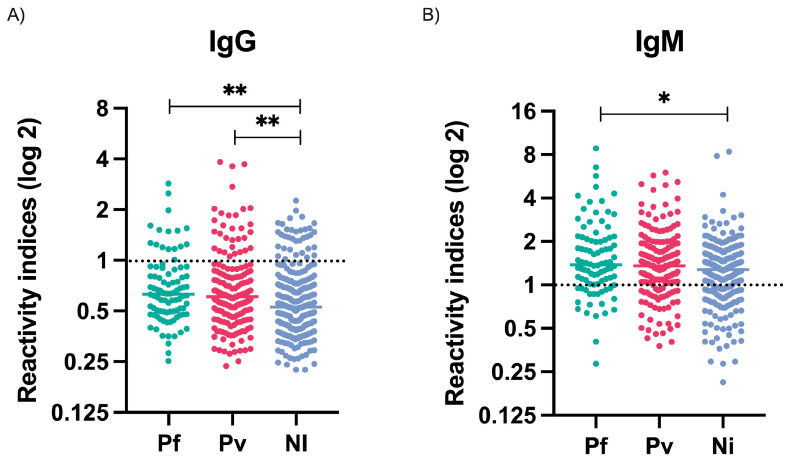
Humoral immune responses to recombinant PfVPS29. (**A**,**B**) Reactivity indices of IgG (**A**) and IgM (**B**) antibodies in non-infected individuals (NI; n = 270) and individuals infected with *P. vivax* (Pv; n = 172) or *P. falciparum* (Pf; n = 90). Individual values are shown. Comparisons were performed using the Kruskal–Wallis test followed by Dunn’s multiple-comparison test with adjusted *p*-values. Dashed lines indicate the cutoff for positivity (reactivity index > 1). Lines represent medians. IgG: ** *p* = 0.0043 (Pv vs. NI), ** *p* = 0.0040 (Pf vs. NI); IgM: * *p* = 0.037 (Pf vs. NI).

When comparing infected and non-infected individuals, no statistically significant difference was observed in the frequency of IgM responders between groups (Table 2). Individuals infected with either *P. vivax* or *P. falciparum* displayed higher IgG antibody levels than non-infected individuals (respectively, *p* = 0.0040, *p* = 0.0043) (Figure 3A). Furthermore, *P. falciparum*-infected individuals exhibited significantly elevated IgM antibody levels when compared to non-infected individuals (Figure 3B).

Comparisons of responder frequencies were performed using Fisher’s exact test. One individual presenting a simultaneous *P. falciparum* and *P. vivax* infection was excluded from this analysis to ensure mutually exclusive comparison groups.

As expected, none of the volunteers in the CO group had detectable antibodies against PfVPS29.

### 3.3. Genetic Diversity of PfVPS29

**Figure 4:**
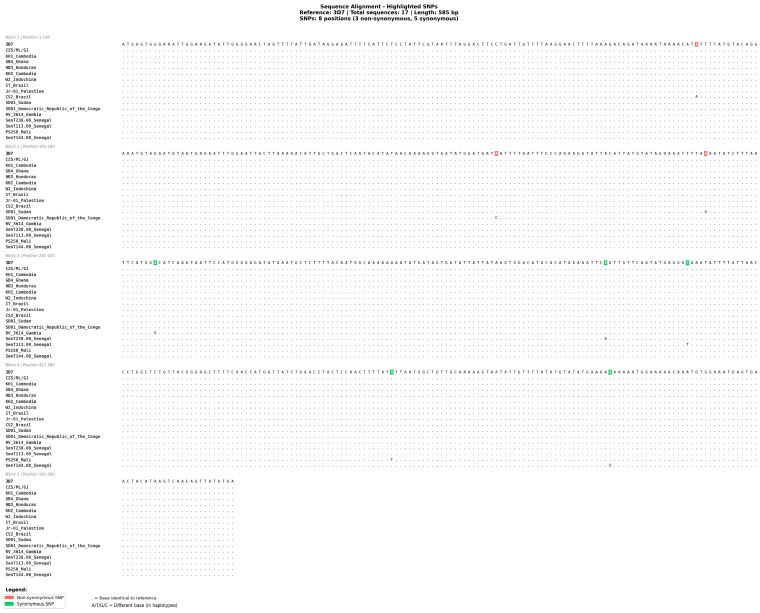
Multiple sequence alignment of PfVPS29 from Brazilian Amazon field isolates and *Plasmodium falciparum* isolates from diverse endemic regions worldwide. The alignment includes 37 sequences (585 bp) from different geographical origins. Non-synonymous single-nucleotide polymorphisms (SNPs) are indicated in red and synonymous SNPs in green. Dots represent nucleotides identical to the reference sequence, whereas letters indicate variant nucleotides in each haplotype.

To investigate the genetic diversity of *vps29*, the full-length gene encoding the PfVPS29 protein was subjected to DNA sequencing in 62 *P. falciparum* isolates from the Brazilian Amazon. This genetic diversity analysis was conducted on a subset of individuals included in the serological survey who presented confirmed *P. falciparum* infection at the time of sampling and for whom parasite DNA of adequate quality and quantity was available. Sequence analysis revealed complete identity with the reference strain 3D7, with no polymorphisms detected in the coding region (Figure 4). Consistent with these findings, BLAST analysis of global VPS29 sequences deposited in GenBank revealed complete identity with the 3D7 reference. Although the PlasmoDB variant catalog (MalariaGEN Pf7 dataset) reports eight single-nucleotide polymorphisms (SNPs) within the *vps29* genomic locus, including three non-synonymous substitutions (G127A, A223G, A269C) and five synonymous substitutions (A288, G387, C405, A480, A528), these variants occur at extremely low global frequencies, typically below detection in geographically restricted sample sets. This likely explains their absence in our dataset and supports the overall high conservation of this gene.

## 4. Discussion

Although the specific function of PfVPS29 has not yet been experimentally characterized in *Plasmodium falciparum*, its high conservation and central role as a core unit of the retromer complex suggest that it is likely important for parasite biology. Despite this, its immunological recognition in naturally exposed human populations has remained largely unexplored, and it has not been traditionally considered a malaria vaccine candidate. Here, we provide the first evidence that PfVPS29 is naturally recognized by the human immune system in individuals chronically exposed to malaria in the Brazilian Amazon.

In spite of living in endemic regions, the cohort displayed substantial heterogeneity in infection history and exposure level—an expected feature of Amazonian populations composed of both native residents and long-term migrants (92% residing in endemic areas for more than 10 years). Such variability is relevant for interpreting naturally acquired immune responses, given that the development of clinical immunity to malaria depends on cumulative and sustained exposure to the parasite [20,21]. Among individuals able to recall infecting species from previous malaria episodes, most reported infections exclusively by *P. vivax* or both *P. vivax* and *P. falciparum*, with *P. vivax* being the most common. This pattern is consistent with the current malaria scenario in Brazil, where nearly 83% of cases are attributed to *P. vivax* [22].

Given that the majority of individuals in our cohort reported previous infections with *P. vivax* alone or in combination with *P. falciparum*, it was essential to assess whether the antibody responses detected against PfVPS29 could be influenced by cross-reactivity with the *P. vivax* ortholog. Sequence comparison between the two proteins revealed approximately 89% identity, indicating high but not complete conservation. This degree of similarity raises the possibility that a fraction of the naturally acquired antibody response could arise from cross-reactive epitopes shared between PfVPS29 and PvVPS29. Although predicted immunodominant linear epitopes of PfVPS29 did not correspond to conserved regions shared with *P. vivax* (Supplementary Table S1), this analysis does not account for conformational epitopes, which may also contribute to antibody cross-reactivity. Therefore, the contribution of cross-reactive antibodies cannot be definitively excluded. Importantly, the epidemiological context of the study area must be considered when interpreting potential cross-reactivity. The population investigated resides in a long-standing malaria hotspot in the western Brazilian Amazon, where *P. falciparum* transmission has historically been intense, and most individuals report previous exposure to both *Plasmodium* species. Among the small subset of individuals who reported living for less than 10 years in endemic areas and exclusive prior *P. vivax* infection, PfVPS29 reactivity was rare and observed only in a single individual who presented a concurrent *P. falciparum* infection at the time of sampling. In contrast, higher reactivity was associated with current or recent *P. falciparum* infection. Together, these observations support an association between PfVPS29 antibody responses and *P. falciparum* exposure. However, in the absence of experimental approaches such as cross-absorption assays, the potential contribution of cross-reactive antibodies derived from prior *P. vivax* exposure cannot be excluded and should be considered a limitation of this study.

Our data indicate that PfVPS29 elicits a weak and infrequent long-term IgG response. These findings are consistent with previous observations showing that naturally acquired antibody responses in malaria are typically directed toward a limited set of surface-exposed or secreted antigens, such as MSP1 and AMA1, whereas responses to highly conserved intracellular proteins tend to be less prominent [23,24]. In contrast, we observed a high prevalence and magnitude of IgM responses to PfVPS29, which may reflect recent or repeated exposure to the parasite, as IgM predominates during early stages of the humoral response [25,26]. Alternatively, PfVPS29 may behave, at least partially, as a T cell–independent (TI) antigen, inducing strong IgM responses with limited class switching to IgG due to insufficient activation of CD4^+^ T cell help. Such responses are often associated with antigens displaying repetitive or highly ordered structures that promote efficient B cell receptor (BCR) cross-linking and pronounced IgM induction [27]. A well-characterized example in malaria is the Circumsporozoite Protein (CSP), whose repetitive NANP motifs drive strong IgM responses with limited or transient IgG production [28]. However, PfVPS29 does not exhibit clear repetitive or multivalent structural features typically associated with classical TI antigens. Therefore, this interpretation remains speculative and is primarily based on the observed IgM-dominated response profile rather than on defined structural properties of the protein.

IgM-dominated responses in malaria are not merely transient or non-functional. Naturally acquired Pf-specific IgM has been shown to contribute to parasite control through complement activation, inhibition of merozoite invasion, and opsonic phagocytosis and has been associated with protection in naturally exposed populations [25,29]. These findings highlight that IgM responses, although often overlooked in favor of IgG, may play a meaningful role in limiting blood-stage parasitemia in malaria. However, the functional activity of PfVPS29-specific IgM antibodies was not evaluated in the present study, and therefore, no conclusions can be drawn regarding their potential contribution to protective immunity.

Nevertheless, because the recombinant PfVPS29 protein was produced in *Escherichia coli* and endotoxin levels were not quantified, we cannot formally exclude a contribution of residual bacterial components to IgM reactivity. While this limitation should be considered when interpreting the magnitude of IgM responses, an important observation argues against non-specific activation as the primary driver of the observed IgM response. If residual bacterial components were responsible for non-specific IgM binding, elevated responses would be expected across all groups, including non-exposed controls, which was not observed. Instead, IgM reactivity was significantly associated with current or recent *Plasmodium falciparum* infection, supporting the interpretation that the observed responses are predominantly antigen-specific rather than driven by non-specific stimulation.

The relevance of the anti-PfVPS29 response becomes particularly noteworthy when considering the biological function of the protein. PfVPS29 is a core component of the retromer complex [12]. Although its specific function has not yet been experimentally defined in *P. falciparum*, evidence from related apicomplexan parasites provides important clues. In *T. gondii*, retromer disruption through TgVps35 knockdown impairs the biogenesis of secretory organelles and affects parasite morphology, demonstrating the essential role of this pathway in apical complex formation [15]. In *P. falciparum*, genome-scale piggyBac studies identified PfVPS29 as refractory to insertions within its encoding region, classifying it as potentially essential for asexual blood-stage in vitro [30]. Although highly speculative, it is plausible that antibodies directed against such a key intracellular complex could exert subtle inhibitory effects, for example, if internalized during parasite invasion. Nonetheless, no evidence currently supports such a mechanism, and this possibility remains hypothetical. Thus, even though PfVPS29 may not constitute a conventional vaccine target, due to its cytoplasmic location and limited IgG response, the immunobiological profile it elicits warrants further investigation as a model of how conserved intracellular parasite proteins are recognized by the human immune system. Taken together, these findings indicate that while IgG responses to PfVPS29 are modest, IgM reactivity is highly prevalent and consistent with patterns previously reported in malaria, although the functional relevance of these responses in the context of PfVPS29 remains to be determined.

Although several correlations reached statistical significance, their low Spearman correlation coefficients indicate weak biological associations. In practical terms, the proportion of individuals mounting an IgG response to PfVPS29 does not increase with cumulative exposure. However, among the subset of individuals who do seroconvert, antibody levels tend to rise only modestly with age and repeated malaria exposure. This pattern, characterized by incremental increases in antibody titers coupled with limited expansion of responder frequency, has been reported for multiple malaria antigens and reflects the slow and inefficient acquisition of humoral immunity in endemic populations [21].

In contrast, IgM responses showed an inverse association with age, duration of residence in endemic areas, and time since the last malaria episode, consistent with higher IgM levels in younger individuals and those with more recent exposure. This pattern supports the interpretation that PfVPS29-specific IgM primarily reflects recent or ongoing parasite exposure rather than durable immunological memory [25]. Importantly, the weak correlation observed between IgG and IgM levels further indicates that these antibody responses are largely dissociated at the individual level.

The dominance of cytophilic IgG1 and IgG3 isotypes is a well-established feature of effective humoral immunity against blood-stage malaria antigens. These subclasses efficiently engage Fc-mediated effector mechanisms, including antibody-dependent cellular inhibition (ADCI) [31,32], opsonic phagocytosis [33], antibody-dependent respiratory burst (ADRB) [34], and complement activation [35,36]. This IgG subclass distribution aligns with the typical immune profile observed for several blood-stage antigens, such as MSP3, GLURP, MSP1, and AMA1 [37,38], for which protective immunity is strongly associated with an IgG1- and IgG3-dominated profile. This suggests that, although PfVPS29 is not a classic immunogenic protein, when an IgG response occurs, it exhibits a subclass profile typically associated with functionally active responses in malaria. In contrast, the non-cytophilic subclasses IgG2 and IgG4 were detected at lower frequencies, in line with previous reports indicating that IgG2 responses may correlate with increased malaria susceptibility, whereas IgG4 is often considered a “blocking” antibody that can interfere with protective mechanisms such as opsonization and antibody-dependent cellular cytotoxicity [37]. Interestingly, IgG2 levels showed a weak positive correlation with time of residence in endemic areas, suggesting that non-cytophilic antibodies may accumulate with prolonged exposure, possibly reflecting chronic immune stimulation or a regulatory adaptation. Taken together, these findings indicate that although PfVPS29 elicits overall modest IgG responses, the subclass distribution tends to shift toward cytophilic antibodies, a profile typically associated with protective responses against blood-stage malaria antigens.

The analysis of antibody responses based on current infection revealed that active infection, particularly with *P. falciparum,* significantly boosts the anti-PfVPS29 IgM response. Individuals infected with *P. falciparum* showed a marked increase in both the frequency of IgM responders and IgM antibody levels compared to non-infected individuals. A similar pattern was observed for IgG levels in infected groups, although the frequency of IgG responders did not differ significantly. These findings align with the understanding that IgM represents an acute and short-lived response directly related to the presence of replicating parasites, likely due to the increased antigenic load and subsequent immune activation. The boost in IgM observed during *P. falciparum* infection could be attributed to the higher parasite densities typically associated with this species compared to *P. vivax*, leading to a greater release of cytoplasmic antigens like PfVPS29 upon schizont rupture, or by the expression of highly immunogenic repetitive motifs in antigens such as CSP, PfEMP1, and MSP1 [39,40], which may favor the IgM response. The increase in IgG levels among infected individuals, despite unchanged responder frequency, suggests that a subset of the population had pre-existing IgG responses to PfVPS29 that were modestly boosted during infection.

The absence of polymorphisms in the *vps29* gene across all Brazilian isolates underscores the remarkable conservation of the PfVPS29 protein in local parasite populations. This finding is consistent with population-level data, although the PlasmoDB variant catalog reports eight SNPs within the vps29 locus, based on the MalariaGEN Pf7 dataset [41]; these variants occur at extremely low global allele frequencies, which likely explains their absence both in our samples and in publicly available GenBank sequences. The high level of conservation is consistent with its essential role as a core component of the retromer complex, which plays a critical role in vesicular trafficking [42]. The lack of genetic variability also suggests that PfVPS29 is not a major target of host immune pressure, since strong immune-driven selection typically results in the emergence of escape variants, as observed for surface-exposed proteins such as AMA1, PfEMP1, and MSP1 [43,44,45]. This is further supported by its intracellular localization, which likely limits the protein’s exposure to the immune system during infection [46].

## 5. Conclusions

In conclusion, this study provides the first characterization of naturally acquired antibody responses to PfVPS29, a highly conserved component of the *P. falciparum* retromer complex. PfVPS29 elicits an atypical profile dominated by IgM and infrequent cytophilic IgG responses. It’s marked conservation, with no polymorphisms in Brazilian isolates and minimal global variability, supports strong functional constraints.

Overall, these findings indicate that PfVPS29 is not a conventional target of naturally acquired immunity, but rather a conserved intracellular protein that elicits limited and atypical humoral responses during malaria exposure.

## Figures and Tables

**Table 1:** Personal and epidemiological characteristics of the individuals enrolled in the survey.

Personal Data		n = 533	CO n = 54
Gender	Male	277/533 (52.4%)	22/54 (40.7%)
	Female	256/533 (47.6%)	32/54 (59.3%)
Age (years)		31 (21–45)	21 (20–24)
Time of residence in malaria-endemic area (years)		29 (20–44)	21 (19–23)
Clinical And Epidemiological Data			
Number of past malaria episodes		10 (3–20)	NA
Time elapsed since the last malaria episode (months)		12 (2–60)	NA
Time of symptoms (days)		4 (2–6)	NA
Diagnosis	*P. falciparum*	90 (16.9%)	NA
	*P. vivax*	173 (32.5%)	NA

Age, time of residence in malaria-endemic areas (years), number of previous malaria episodes, time elapsed since the last malaria episode (months), and duration of symptoms (days) are expressed as median and interquartile range (IQR). n: number of individuals; %: percentage, CO: control group.

**Table 2:** IgG and IgM antibody responses in non-infected individuals and individuals infected with *Plasmodium vivax* or *Plasmodium falciparum*.

		NI	PV	PF
IgM	Responder	170/270 (63%)	109/172 (63.4%)	65/90 (72.2%)
	Non-responder	100/270 (37%)	63/172 (36.6%)	25/90 (27.8%)
		**NI**	**PV**	**PF**
IgG	Responder	35/270 (13%)	28/172 (16.3%)	17/90 (18.9%)
	Non-responder	235/270 (87%)	144/172 (83.7%)	73/90 (81.1%)

NI: non-infected individuals; PV: *P. vivax*-infected individuals; PF: *P. falciparum*-infected individuals.

## Data Availability

The datasets supporting the conclusions of this article are included within the article and its Supplementary Materials.

## Supplementary Materials

The following supporting information can be downloaded at: https://www.mdpi.com/article/10.3390/pathogens15070691/s1, Figure S1: PFVPS29 expression and purification results; Table S1: Antigenicity prediction of the sequences; Table S2: Comparison of identity between predicted epitopes and previously described epitopes in the literature.
