# Supplementary material for: Antibody Responses to the Conserved Plasmodium falciparum Vacuolar Sorting Protein 29 in the Brazilian Amazon
Source: Pathogens. 2026 Jun 30;15(7):691. doi: 10.3390/pathogens15070691 (PMC13414709; doi:10.3390/pathogens15070691)
Supplement: Supplementary file 1 [file pathogens-15-00691-s001.zip › Suplemmentary Table S1.pdf]

Table S1: Antigenicity prediction of the sequences.

Epitope	Sequence	Lenght	Vaxijen Score
$VPS29_{(48-59)}$	NVGCNENLELLK	12	0,2527
$VPS29_{(68-79)}$	TKGDMDDNFDFP	12	0,7007
$VPS29_{(111-118)}$	WQKKYDSD	8	0,5908
$VPS29_{(150-157)}$	PWLSEPTP	8	0,8665
$VPS29_{(173-184)}$	VYEEKNGKTNVE	12	1,0988

Predicted linear epitopes in the PfVPS29 sequence using the VaxiJen v2.0 server. The corresponding positions in the sequence (in parentheses), peptide sequences, amino acid lengths, and VaxiJen scores—indicating antigenic potential—are shown. Values above 0.5 are considered indicative of probable antigenicity. The only non-antigenic epitope is highlighted in red.

Table S2: Comparison of identity between predicted epitopes and previously described epitopes in the literature.

Epitope / Protein	Sequence	Identity
$VPS29_{(68-79)}$	TKGDMDDNFDFP	
PfMSP9	TVSGMDENFDNH	50%
Onchocerca_volvulus	RCYLMDDNYDNL	41,67%
$VPS29_{(111-118)}$	WQKKYDSD	
Thioredoxin domain-containing protein 9	RGKKYDSD	75%

In the table, the letters represent amino acids in the one-letter code. Predicted epitopes are shown in black, whereas sequences of epitopes previously described in the literature display conserved amino acids in blue and differing amino acids in red. The degree of identity between the predicted epitope and the previously described sequence is expressed as a percentage in the table.
