# Supplementary figures and images for: Antibody Responses to the Conserved Plasmodium falciparum Vacuolar Sorting Protein 29 in the Brazilian Amazon
Source: Pathogens. 2026 Jun 30;15(7):691. doi: 10.3390/pathogens15070691 (PMC13414709; doi:10.3390/pathogens15070691)

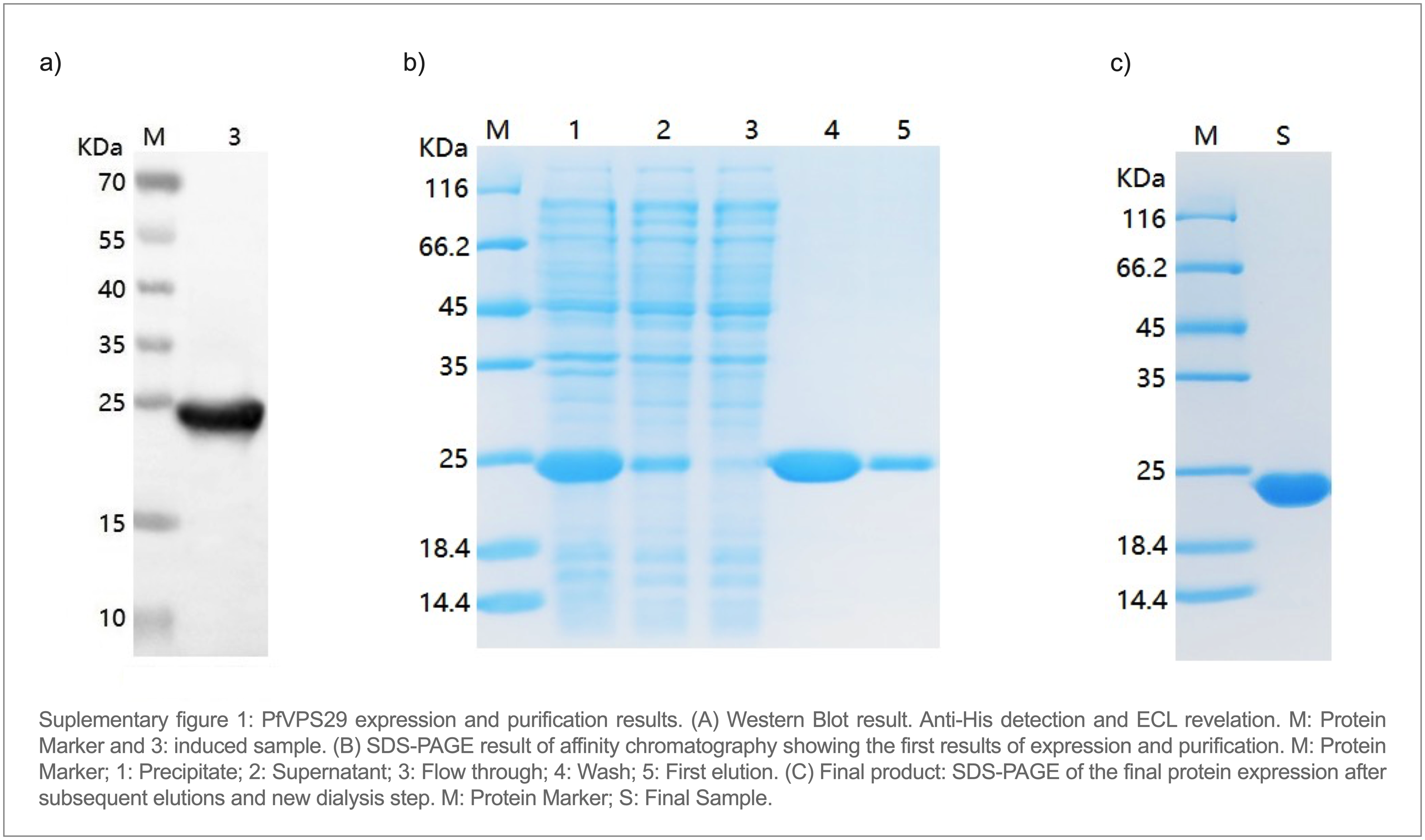

Supplement: Supplementary file 1 [file pathogens-15-00691-s001.zip › Supplementary Figure S1.tiff]
